# Awareness of social functioning in people with dementia and its association with dementia severity: multi-centre cross-sectional study

**DOI:** 10.3233/JAD-240311

**Published:** 2024-01-01

**Authors:** Andrew Sommerlad, Jessica Grothe, Sumiyo Umeda, Manabu Ikeda, Hideki Kanemoto, Gill Livingston, Melanie Luppa, Katherine P. Rankin, Steffi G. Riedel-Heller, Susanne Röhr, Maki Suzuki, Jonathan Huntley

**Affiliations:** 1 Division of Psychiatry, University College London, Gower Street, London WC1E 6BT, UK; 2 Camden and Islington NHS Foundation Trust, St Pancras Hospital, London, UK; 3 Institute of Social Medicine, Occupational Health and Public Health (ISAP), Medical Faculty, University of Leipzig, Germany; 4 Department of Psychiatry, Osaka University Graduate School of Medicine, Osaka, Japan; 5 Department of Psychiatry, Daini Osaka Police Hospital, Osaka, Japan; 6 Department of Psychiatry and Mental Health, Sumitomo Hospital, Osaka, Japan; 7 Health and Counseling Center, Osaka University, Osaka, Japan; 8 Department of Neurology, Memory and Aging Center, University of California, San Francisco, USA; 9 Global Brain Health Institute (GBHI), Trinity College Dublin, Dublin, Ireland; 10 School of Psychology, Massey University, Albany Campus, Auckland, Aotearoa New Zealand; 11 Department of Behavioral Neurology and Neuropsychiatry, Osaka University United Graduate School of Child Development, Osaka, Japan; 12 The Medical School, University of Exeter, Exeter, UK

**Keywords:** Alzheimer’s disease, dementia, social functioning, insight, awareness, metacognition

## Abstract

**Background:**

People with dementia commonly have impaired social functioning and may not recognise this. This lack of awareness may result in worse outcomes for the person and their family carers.

**Objective:**

We aimed to characterise awareness of social functioning in dementia and describe its association with dementia severity.

**Methods:**

Multi-centre cross-sectional study of people aged >65 years with dementia and family informants recruited from Germany, Japan and the United Kingdom. We used the Social Functioning in Dementia (SF-DEM) scale, assessing “spending time with other people” (domain 1), “communicating with other people” (domain 2), and “sensitivity to other people” (domain 3), and calculated lack of awareness into social functioning as the discrepancy between patient and informant ratings.

**Results:**

108 participants with dementia (50.9% women), mean age = 78.9 years, and mean MMSE score = 22.7. Patient and informant domain 1 ratings did not differ, but patient-rating was higher than carers for domain 2 (11.2 v 10.1; p = 0.003) and domain 3 (9.7 v 8.1; p < 0.001). Sixty people with dementia overestimated their overall social functioning, 30 underestimated, and 18 gave ratings congruent with their informant. Performance on the MMSE and its sub-domains was not associated with SF-DEM discrepancy score.

**Conclusions:**

We found that awareness of social functioning in dementia was a multidimensional concept, which varies according to subdomains of social functioning. Clinicians should help family members understand and adapt by explaining their relative with dementia’s lack of awareness about aspects of their social functioning.

## Introduction

Impairment of social functioning is characteristic of dementia ^1^ and troubling for people with dementia and their families. Impaired awareness of cognition or level of function is also characteristic of dementia. ^2^ People with dementia often overestimate their ability, perceiving their abilities to be maintained when they have deteriorated. ^3^ This impaired awareness is associated with structural and functional abnormalities in multiple brain regions including the inferior frontal gyrus, anterior cingulate cortex, and medial temporal lobe. ^4^ Lack of awareness is associated with worse outcomes including functional impairment and deteriorating dementia severity, ^5^ neuropsychiatric symptoms including higher levels of apathy, psychosis, and anxiety, ^5, 6^ and worse carer outcomes ^5, 7^ and may potentially affect the safety of people with dementia as they perceive the risk linked to their behaviour inaccurately.

People with dementia may also lack awareness of impairments of their social functioning, often overestimating their ability. ^3, 8–15^ This overestimation of social functioning has been associated with infero-lateral temporal lobe atrophy. ^16, 17^ However, studies have been limited by small sample sizes, ^11–14^ and used instruments with uncertain validity which do not differentiate between different components of social functioning. ^8–10, 13^

Previous research has used several approaches to assess awareness of any domain of cognition or functioning including 1) self-appraisal of performance, where a person with dementia judges their performance on an assessment and this prediction is compared with their objective score; 2) informant rating, where a clinician, relative or friend judges the awareness of the person with dementia; or 3) patient-informant discrepancy scores, where assessments of cognition or function are completed by both the patient and a knowledgeable informant and the discrepancy between the scores is used as a measure of awareness, which assumes that the appraisal of a knowledgeable non-cognitively-impaired informant’s will be accurate ^18^. Accurate assessment of awareness into social functioning in dementia has been hampered by a lack of validated tools to assess social function. However, the social functioning in dementia (SF-DEM) scale is a valid and reliable instrument for assessing different aspects of social functioning in people with dementia, ^19^ which has been translated into several languages and culturally adapted and validated in these settings, ^20, 21^ and has patient and carer rated versions allowing exploration of rating discrepancies. ^22^

We therefore aimed to characterise awareness into social functioning in an international multi-centre study of people with mild and moderate dementia, and test our hypothesis that impaired awareness would be associated with more severe cognitive impairment.

## Methods

### Study design

Observational cross-sectional study of people with dementia and family informants recruited from three study sites in Germany, Japan and the United Kingdom (UK). Ethical approval for the study was from the Ethics Committee of the Medical Faculty of the University of Leipzig (ref: 401/19-ek) for Germany; the Ethical Committee of Osaka University Hospital (No. 200305) and Daini Osaka Police Hospital for Japan, and the Westminster NRES Committee (15/LO/0105) for the UK.

### Setting and participants

We included people aged 65 years and over with clinically-diagnosed dementia. Participants from Germany were recruited if they met International Statistical Classification of Diseases: 10th revision (ICD-10) dementia criteria; ^23^ participants from Japan were recruited if they met the criteria for dementia from the National Institute on Aging and Alzheimer’s Association (NIA-AA); ^24^ UK participants were required to meet dementia criteria from the Diagnostic and Statistical Manual of Mental Disorders, Volume 5 (DSM-V). ^25^ Dementia could be of any subtype and participants were not required to have biomarker-guided diagnosis. Participants were included if they had mild to moderate dementia severity, indexed by a mini-mental state examination ^26^ score 11 or higher, which has substantial agreement with other ratings of dementia severity. ^27^ We required an available informant who was a relative or close friend aged over 18 years who saw the person with dementia at least weekly to be able to provide an accurate appraisal of their level of social functioning. We excluded people with severe dementia, those with severe physical or mental illness which would limit their participation in the interviews, and those who were unable to give informed consent.

#### Germany

We recruited participants from the memory day clinic of University Hospital of Leipzig, a contact point for people with memory impairment that offers comprehensive diagnostics, therapy and treatment.

#### Japan

We recruited participants from an outpatient clinic for dementia in Department of neuropsychiatry of Osaka University Hospital and Department of Psychiatry and Neurology of Daini Osaka Police Hospital.

#### UK

We recruited participants from the memory clinics in a North London-based National Health Service secondary mental healthcare trust, Camden and Islington NHS Foundation Trust, which provides dementia assessment and treatment services.

### Measures

#### Social functioning

The Social Functioning in Dementia scale (SF-DEM) ^19^ is a 20-item interviewer-administered questionnaire, which has patient- and carer-rated versions. Seventeen items about different aspects of social function are scored using a Likert scale (0 to 4 indicating frequency of each social function domain; “never” to “very often”) with a higher score indicating better social function. The 17 items cover different aspects of social functioning which cover three independent domains: “spending time with other people” (Domain 1), “communicating with other people” (Domain 2), and “sensitivity to other people” (Domain 3). ^28^ The German ^21^ and Japanese ^20^ translations of the original English version have been validated. The minimum clinically important difference, meaning the smallest change or difference in an outcome measure that is perceived as beneficial, is 1.9 points for domain 1, 2.0 points for domain 2 and 1.4 points for domain 3 or, if applied to an individual patient, 2 points for each domain. ^29^

We calculated continuous discrepancy scores between patient and informant ratings for each SF-DEM domain by subtracting the informant rated-score from the patient-rated score, meaning that positive scores indicate patients’ overestimation of their functioning, and negative scores indicates underestimation, with higher scores indicating greater overestimation. We also categorised the discrepancy scores into overestimation (≥ 2), congruent (+1 to -1), underestimation (≤ -2). We chose the threshold of 2 points discrepancy as indicating meaningful discrepancy as this is the minimum clinically meaningful difference for each SF-DEM domain for individual patients. ^29^

#### Other characteristics

We collected information on age, sex, marital status (married/common law partner, single/divorced/separated, widowed), and living status (living alone, with others) of the person with dementia, and their level of education (primary/lower secondary, higher secondary, graduate/postgraduate). Cognition was assessed by the mini-mental state examination (MMSE) in German, Japanese or English language, which includes domains assessing orientation to time and place, registration and subsequent delayed recall of a list of three words, attention using a basic calculation test, language and repetition and ability to follow complex commands. ^26^ Dementia subtype was ascertained from clinical notes and checked against diagnostic criteria (NIA-AA ^24^ and McKeith criteria for probable AD and DLB respectively in Japan, and DSM-V criteria in UK. ^25^ Severity of dementia was indexed by MMSE score (≥ 20 points = mild dementia, < 20 and ≥ 10 = moderate). ^27^ We recorded the age and sex of the family informant and their relationship with the person with dementia.

### Statistical analyses

We first described socio-demographic data for all participants and for each site, using chi-squared test and t-tests to compare these characteristics between sites. Thereafter, data were combined between the three sites with combined data used as the primary results. We also reported results separately for each site.

We described the continuous and categorical discrepancy scores for each SF-DEM domain and the full SF-DEM for the three sites combined and for each site individually, using paired t-test to compare the ratings of the patient and carer, at significance level p=0.05. We then calculated the association of the SF-DEM discrepancy score for each domain with severity of dementia using linear regression, whereby the coefficient reflects the number of SF-DEM points overestimation by the patient compared to the carer per 10 point worse performance on MMSE. We present unadjusted results and results adjusted for age, sex, whether the person with dementia lived alone or with the informant, and study site. In a post-hoc secondary analysis, we calculated SF-DEM discrepancy scores for each domain across different dementia subtypes.

We then calculated the association of awareness into social functioning (categorical overestimation, congruent, underestimation) with dementia severity, using multinomial logistic regression whereby the relative-risk ratio (RRR) reflects the risk of the patient being an over- or under-estimator compared to their family informant, per 10 point worse MMSE performance. We hypothesised that impaired awareness may reflect deficits in the specific cognitive domains of recall or attention, because people with dementia may not have encoded memory of recent events. We therefore examined whether SF-DEM discrepancy score was associated with performance on the MMSE sub-domains testing delayed recall and attention / calculation, using linear regression whereby the coefficient reflects the number of SF-DEM points overestimation by the patient compared to the carer per one point worse performance on each MMSE subdomain, unadjusted and adjusted for age, sex, living status, and study site.

## Results

We obtained data from 108 people with dementia (29 in Germany, 49 in Japan and 30 in the UK) and their and their informants’ sociodemographic and clinical characteristics are summarised (Table 1). The mean age of the people with dementia was 78.9 (standard deviation (SD) 6.5) years, 55 (50.9%) were women, and 76 (70.4%) lived with others with the remainder living alone. Mean MMSE score was 22.7 (SD 3.7), 86 (79.6%) had mild dementia, and for around half of participants (56, 51.9%) the dementia subtype was Alzheimer’s disease. The informants’ mean age was 66.8 (SD 13.1) and 72 (66.7%) were women. Sixty of the informants (55.6%) were spouses and 41 (38.0%) were a child of the person of dementia. There were differences across the sites in the person with dementia’s level of education (low educational level more frequent in UK participants), their dementia severity (more severe in German participants and milder in UK, and subtype (Lewy body dementia more frequent in Japan and dementia subtype unknown in German participants), and informant characteristics.

### SF-DEM scores and discrepancies

**Figure 1:**
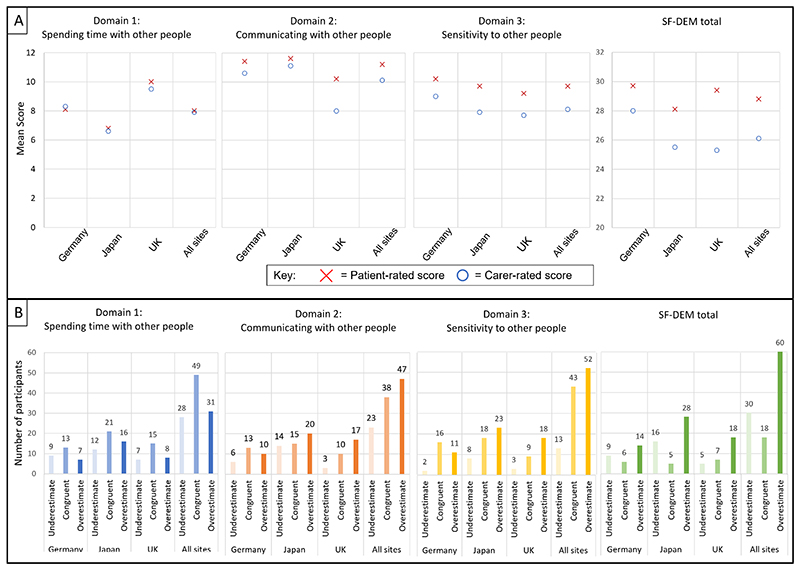
(A) Mean SF-DEM scores rated by patients and carers, and (B) discrepancies between patient and carer ratings

The scores for each SF-DEM domain as rated by patients and carers in each study site and for all participants are shown (Figure 1, Supplementary Table 1, Supplementary Table 2. There was no significant difference between the ratings of patients and carers for any of the study sites for domain 1, with the mean patient-rated score 8.0 (SD 2.5) and mean carer-rating 7.9 (SD 2.8) (p = 0.58, t = 0.55), across all sites. Patient-rating was higher than carer-rating for domain 2 in all sites with a statistically significant higher rating by patients in the UK, and across all sites (patient-rated score 11.2 (SD 2.5), carer-rated score 10.1 (3.4); p = 0.003, t = 2.98). For domain 3, patient-rating was higher than the carer-rating in all study sites and in combined results (patient-rated score 9.7 (SD 2.4), carer-rated score 8.1 (SD 2.8); p < 0.001, t = 4.98). The mean patient-rating of the overall SF-DEM was 28.8 (SD 4.6) and the carer-rating was 26.1 (SD 5.5; p < 0.001, t = 5.19).

Twenty-eight (25.9%) people with dementia underestimated their domain 1 social functioning (“spending time with others”) compared with their family informant, 49 (45.4%) of ratings were congruent with the family carer, and 31 (28.7%) overestimated their time spent with others. For domain 2 (“communicating with others”), 23 (21.3%) of people with dementia underestimated, 38 (35.2%) were congruent, and 47 (43.5%) overestimated. For domain 3 (“sensitivity to others”), the number of people with dementia who underestimated, were congruent, and overestimated were 13 (12.0%), 43 (39.8%) and 52 (48.2%) respectively. For the overall SF-DEM rating, over half of people with dementia overestimated their overall social functioning (60, 55.6%), 30 (27.8%) underestimated, and 18 (16.7%) gave ratings which were congruent with their family informant.

We report discrepancy scores by dementia subtype (Supplementary Table 3), finding greater over-estimation of social functioning in people with dementia caused by Alzheimer’s disease (3.5 (SD 5.8)) compared to dementia with Lewy bodies (0.4 (SD 5.1), p=0.04). People with vascular dementia had the highest overestimation (7.8 (SD 4.8)) but this was a small sample of 5 participants.

### Association of awareness with dementia severity

Participants with more severe dementia (per 10 points worse MMSE performance) did not have a significantly different discrepancy between self- and carer-rated SF-DEM in any domain (Table 2). For each 10 point worse MMSE performance, the patient-rating was 0.7 points higher (95% confidence interval -1.1, 2.5) than the carer-rating for domain 1 (i.e. more likely to overestimate their time spent with others than a participant with milder dementia). Rating of patients with more severe dementia was 0.6 points lower (95% CI -3.1, 1.9) than the carer-rating for domain 2, and 1.6 points higher for domain 3 (95% CI -0.5, 3.8). The overall SF-DEM rating was 1.7 points higher (95% CI -1.9, 5.4) for patient-rating compared to carer ratings for patients with more severe dementia.

Having more severe dementia was not associated with elevated risk of underestimating or overestimating social functioning compared to those with milder dementia (Table 2). Worse performance on MMSE by 10 points was associated with RRR of 0.64 (95% CI 0.07, 5.73) for underestimating total social functioning compared to giving a rating congruent with the family informant. RRR for being an over-estimator compared to giving congruent ratings was 0.86 (95% CI 0.12, 6.15)

Performance on the MMSE domains of recall, or attention/calculation was not significantly associated with SF-DEM discrepancy score (Supplementary Table 4). For each one point worse performance on the recall scale, patients rated their total SF-DEM 0.3 (95% CI -0.8, 1.4) points higher than their informant. And for each point worse on the attention/calculation subdomain, patients overestimated total SF-DEM score by 0.4 (95% CI -0.3, 1.0) points.

## Discussion

In this study of 108 people with dementia across three countries, we found that awareness of social functioning, indexed by the discrepancy between the rating of the person with dementia and that of a knowledgeable family informant, varied according to the domain of social functioning being rated. Awareness was impaired in communication with other people and sensitivity to other people. However, ratings of the amount of time spent with other people were congruent between the person with dementia and their family carer in around half of cases, with around one quarter overestimating and one quarter underestimating the amount of time together. There was no statistically- or clinically-significant difference between the mean ratings of people with dementia and their family carers. In contrast, in ratings of the level of communication of the person with dementia or their sensitivity to other people, it was most common for the person with dementia to overestimate their level of functioning, compared to their family informant. The mean ratings for patients and carers differed significantly with the mean discrepancy for the sensitivity to other people also exceeding the minimum clinically-significant difference. We found that poor awareness was not associated with worse dementia severity and this suggests it is not a function of impaired memory.

The domains where we found discrepancy between patient and carer ratings required individuals to estimate the quality and nature of their interaction with another person. For example in the ‘Communicating with other people’ domain, we asked ‘Thinking about the past month, how often have you asked other people about their feelings or concerns?’ or in the ‘Sensitivity to other people’ domain we asked ‘Thinking about the past month, how often have you had an argument or shouted at other people?’. Awareness was intact for our study participants in the ‘Spending time with other people’ domain of social functioning, in which questions were asked about frequency of contact with other people, e.g. ‘Thinking about the past month, how often have you seen family or friends in your own home?’ Accurate response to all these questions requires participants to recall the frequency of past behaviours but the domains which were impaired additionally require subjective judgement about the nature of the behaviour, for example whether an interaction could be defined as an argument, or whether a conversation could be described as enquiring about another person’s feelings. We found that the domains with greater subjectivity were impaired whereas the more objective domains were not.

These results were largely consistent across the research sites, particularly for domains 1 and 3, despite some differences in the demographic and clinical characteristics of participants between the countries. For domain 2, UK participants overestimated their functioning more than participants from other countries, which may reflect socio-cultural differences in expectations of people with dementia and their informants about social communication. The discrepancy score was slightly lower for German participants despite them having worse dementia severity although the pattern across the three SF-DEM domains was consistent with that of other sites.

Several studies have suggested the level of awareness is contingent on the domain of awareness. For example two UK studies of people with mild dementia found that awareness into level of socio-emotional function – assessed using the socio-emotional questionnaire (SEQ) which assesses 30 aspects of social cognition and behaviour – was not correlated with awareness into memory or activities of daily living. ^7, 30^ Another Brazilian study of 89 people with Alzheimer’s disease using the discrepancy between patient and carer ratings on the Assessment Scale of Psychosocial Impact of the Diagnosis of Dementia (ASPIDD) scale ^31^ as an index of awareness of social functioning and relationships found that awareness for this domain was not associated with awareness into other aspects of functioning or cognition. ^9^

Other studies ^8–10^ have reported lower level of impairment on the ASPIDD subscales for social functioning/relationships (range 1 to 1.3) compared to the subscales for cognition (range 1 to 3.3) or functional activity impairment (range 2 to 5.7). However, these scores are not directly comparable as the scales differ in their score ranges, and the scoring of the ASPIDD only refers to the number of discrepant ratings but does not describe whether the person with dementia over- or under-estimated their level of function. The SEQ examines awareness of the social cognition and behaviour domains of emotion recognition and empathy, social relationships and prosocial behaviour, which are different from the SF-DEM’s assessment of social functioning and MCID is not established. Our study adds to the literature by clarifying the proportion of people who underestimate and overestimate social functioning, and by using a scale which has established MCID allowing determination of whether differences in different subscales are clinically meaningful. We also report preliminary findings suggesting that awareness of social functioning is more impaired in Alzheimer’s disease and vascular dementia than dementia with Lewy bodies, and note that previous studies have reported inconsistent findings on awareness in these dementia subtypes. ^32^

Our finding that level of awareness was not associated with overall dementia severity or impairment of recall or attention, was contrary to our hypothesis. Previous findings suggested that the SF-DEM overall had lower validity for self-rating in people with dementia whose MMSE score was below 25, compared to those with MMSE ≥25, and found that awareness of social functioning declined with more severe dementia and impaired cognition. ^14^ The lack of association found in our study may reflect the small sample size and small variation in dementia severity within our sample with 80% having mild dementia. As we indexed awareness by the difference between self- and carer-rating, it may also be that carer ratings are less reliable as dementia progresses, whereby family members have not adjusted their rating as dementia progresses and so family ratings, like self-ratings tend to become overestimated in more severe dementia. However, our finding is also consistent with two other studies which did not find that awareness of socio-emotional functioning declines with more severe dementia rated by clinical dementia rating (CDR) scale, whereas awareness of cognition and general functional activity was associated with dementia severity. ^9, 10^ Another study suggested that the relationship between awareness and cognition is non-linear, whereby awareness is relatively preserved for MMSE scores above 23, and only declines with MMSE scores <23. ^33^

### Strengths and limitations

Our study has several strengths including the international sample which gives our findings generalisability at least in high income countries, use of an validated instrument ^19^ which has been translated using gold-standard approaches. ^21^ However, the study has limitations. The cross-sectional study design and relatively small number of people with moderate to severe dementia will have impacted our ability to examine changes in awareness with more severe dementia. Awareness is a complex concept and discrepancy scoring using informant ratings may not always be accurate, particularly for those informants who see their friend or relative with dementia less frequently, ^34^ so triangulating discrepancy scores with other approaches to assessing awareness, such as task-based assessments, ^35^ would be beneficial. We lacked information about other dimensions which could have informed our results, such as neuropsychological performance, mood and anxiety and due to small numbers and a lack of clinical information, it was also not possible to clarify which factors may be relevant to whether a person with dementia over or underestimates their social functioning. Although our study did not find evidence that dementia severity relates to lack of awareness, it may be that dementia subtype, or other factors such as mood and anxiety may be relevant to whether a person, particularly with mild dementia may over or underestimate their social functioning. We lacked sufficient sample size to examine results by dementia subtype so cannot draw strong conclusions about whether awareness varies by disease.

### Clinical implications and future research

The findings from this study that awareness varies according to subdomains of social functioning indicate the need for researchers and clinicians to treat awareness as a multidimensional, rather than a single unified, concept. Researchers obtaining information about social functioning in people with mild dementia can use self-rating of objective domains, such as time spent with other people, as these are likely to be accurate, but caution is needed for self-ratings of other domains. Clinicians can inform family members about the lack of awareness of their relative with dementia into some aspects of their socio-emotional functioning and a nuanced understanding of these symptoms may help them to understand and adapt. ^36^ Future research could consider the neural correlates of awareness into social functioning and extend this study to higher numbers of those with moderate and severe dementia, as well as examine in more detail whether specific dementia subtypes have differential levels of awareness.

## Supplementary Material

Supplementary Tables

## Figures and Tables

**Table 1:** Sociodemographic and clinical characteristics of participating dyads

Characteristics			All participants (n = 108)		Germany (n = 29)		Japan (n = 49)		UK (n = 30)		Comparison of research sites
			n	%	n	%	n	%	n	%	
**People** **with** **dementia** **(n=108)**	Age	mean (SD)	78.9 (6.5)		77.9 (4.8)		79.1 (6.3)		79.5 (8.2)		t=0.90, p=0.37
	Sex	Female	55	50.9	12	41.4	28	57.1	15	50	X^2^ = 1.8, p=0.40
		Male	53	49.1	17	58.6	21	42.9	15	50	
	Education	Primary / lower secondary	32	29.6	2	6.9	11	22.5	19	63.3	X^2^ = 76.8, p<0.01
		Higher secondary	46	42.6	24	82.8	21	42.9	1	3.3	
		Graduate / postgraduate	30	27.8	3	10.3	17	34.7	10	33.3	
	Living status	With others	76	70.4	20	69.0	36	73.5	20	66.7	X^2^ = 0.45, p=0.80
		Alone	32	29.6	9	31.0	13	26.5	10	33.3	
	MMSE score	mean (± SD)	22.7 (3.7)		19.3 (3.9)		22.8 (1.7)		25.8 (3.1)		t=0.90, p<0.01
		Mild (30-21)	86	79.6	11	37.9	49	100	26	86.7	X^2^ = 44.8, p<0.01
		Moderate (20-11)	22	20.3	18	62.1	0	0	4	13.3	
	Dementia subtype	Alzheimer’s disease	56	51.9	0	0	34	69.4	22	73.3	X^2^ = 13.3, p<0.01
		DLB	17	15.7	0	0	15	30.6	2	6.7	
		Vascular dementia	5	4.6	0	0	0	0	5	16.7	
		Unspecified dementia	30	27.8	29	100	0	0	1	3.3	
**Informants** **(n=108)**	Role of informant	Spouse/partner	60	55.6	23	76.7	22	44.9	15	50.0	X^2^ = 15.0, p=0.02
		Child	41	38.0	7	23.3	24	49.0	11	36.7	
		Other relative	5	4.6	0	0	3	6.1	2	6.7	
		Friend	2	1.9	0	0	0	0	2	6.7	
	Age of informant	mean (SD)	66.8 (13.1)		71.1 (11.1)		65.7 (14.0)		64.9 (12.9)		t=-1.8, p=0.07
	Sex of informant	Female	72	66.7	13	44.8	35	71.4	24	80	X^2^ = 9.1, p=0.01
		Male	36	33.3	16	55.2	14	28.6	6	20	

Key: DLB = Dementia with Lewy bodies; MMSE = mini-mental state examination; SD = standard deviation

**Table 2:** Association of awareness of social functioning with dementia severity

			Model 1: unadjusted (n=108)			Model 2: adjusted for age, sex, living alone, site, (n=108)		
			Coefficient	95% CI	p-value	Coefficient	95% CI	p-value
Number of SF-DEM points overestimation by patient per ten points worse MMSE performance	**Spending time with other people**		-0.2	-1.5, 1.2	0.80	0.7	-1.1, 2.5	0.46
	**Communicating with other people**		-1.2	-3.1, 0.7	0.23	-0.6	-3.1, 1.9	0.65
	**Sensitivity to other people**		0.6	-1.1, 2.3	0.50	1.6	-0.5, 3.8	0.14
	**SF-DEM Total**		-0.8	-3.6, 2.1	0.59	1.7	-1.9, 5.4	0.35
			**RRR**	**95% CI**	**p-value**	**RRR**	**95% CI**	**p-value**
Risk of patient rating being over- or underestimate per ten points worse MMSE performance	**Spending time** **with other** **people**	**Underestimate**	1.66	0.48, 5.78	0.43	1.21	0.22, 6.57	0.83
		**Congruent (ref)**	Ref			Ref		
		**Overestimate**	0.91	0.26, 3.19	0.89	0.79	0.15, 4.16	0.78
	**Communicating** **with other** **people**	**Underestimate**	0.80	0.20, 3.17	0.75	0.43	0.06, 2.97	0.39
		**Congruent (ref)**	Ref			Ref		
		**Overestimate**	0.28	0.08, 0.99	0.05	0.27	0.05, 1.40	0.12
	**Sensitivity to** **other people**	**Underestimate**	0.19	0.03, 1.29	0.09	0.15	0.01, 2.10	0.16
		**Congruent (ref)**	Ref			Ref		
		**Overestimate**	0.43	0.14, 1.35	0.15	0.99	0.22, 4.53	0.99
	**SF-DEM Total**	**Underestimate**	1.58	0.32, 7.75	0.57	0.64	0.07, 5.73	0.69
		**Congruent (ref)**	Ref			Ref		
		**Overestimate**	0.99	0.23, 4.23	0.99	0.86	0.12, 6.15	0.88

Key: CI = confidence interval; MMSE = mini-mental status examination; ref = reference; RRR = Relative risk ratio; SF-DEM = Social functioning in dementia scale

## Data Availability

The data supporting the findings of this study are available on request from the corresponding author. The data are not publicly available due to privacy or ethical restrictions.
