# Supplementary material for: Awareness of social functioning in people with dementia and its association with dementia severity: multi-centre cross-sectional study
Source: J Alzheimers Dis. Author manuscript; Available in PMC 2025 Aug 16. (PMC7618018; doi:10.3233/JAD-240311)
Supplement: Supplementary Tables [file EMS207652-supplement-Supplementary_Tables.pdf]

Supplementary Table 1: SF-DEM scores and discrepancies between patient and informant ratings

Characteristics		Patient-rated mean (SD) min, max	Carer-rated mean (SD) min, max	t-test
**Spending time with others**	Germany	8.1 (2.5)<br>2, 12	8.3 (2.7)<br>3, 12	p = 0.52<br>t = -0.65
	Japan	6.8 (2.8)<br>2, 13	6.6 (2.4)<br>3, 12	p = 0.60<br>t = 0.52
	UK	10.0 (2.5)<br>5, 15	9.5 (2.7)<br>4, 15	p = 0.40<br>t = 0.85
	All sites	8.0 (2.9)<br>2, 15	7.9 (2.8)<br>3, 15	p = 0.58<br>t = 0.55
**Communicating with others**	Germany	11.4 (2.4)<br>7, 16	10.6 (3.0)<br>5, 17	p = 0.22<br>t = 1.26
	Japan	11.6 (2.6)<br>6, 17	11.1 (3.3)<br>4, 18	p = 0.37<br>t = 0.91
	UK	10.2 (2.3)<br>4, 17	8.0 (3.3)<br>0, 14	p < 0.001<br>t = 3.90
	All sites	11.2 (2.5)<br>4, 17	10.1 (3.4)<br>0, 18	p = 0.003<br>t = 2.98
**Sensitivity to others**	Germany	10.2 (2.2)<br>3, 12	9.0 (1.9)<br>5, 12	p = 0.008<br>t = 2.86
	Japan	9.7 (2.5)<br>1, 12	7.9 (3.2)<br>0, 12	p = 0.001<br>t = 3.34
	UK	9.2 (2.5)<br>3, 12	7.7 (2.7)<br>2, 12	p = 0.01<br>t = 2.61
	All sites	9.7 (2.4)<br>1, 12	8.1 (2.8)<br>0, 12	p < 0.001<br>t = 4.98
**Total SF-DEM**	Germany	29.7 (4.2)<br>18, 36	28.0 (4.4)<br>19, 36	p = 0.06<br>t = 1.96
	Japan	28.1 (4.2)<br>19, 37	25.5 (5.5)<br>10, 35	p = 0.003<br>t = 3.08
	UK	29.4 (5.4)<br>18, 38	25.3 (6.2)<br>16, 36	p = 0.001<br>t = 3.94
	All sites	28.8 (4.6)<br>18, 38	26.1 (5.5)<br>10, 36	p < 0.001<br>t = 5.19

Key: max = maximum, min = minimum, SD = standard deviation

Supplementary Table 2: SF-DEM scores: mean discrepancies between patient and informant ratings

Characteristics			All participants (n = 108)	Germany (n = 29)	Japan (n = 49)	UK (n = 30)
			**mean (SD)** **min, max**	**mean (SD)** **min, max**	**mean (SD)** **min, max**	**mean (SD)** **min, max**
**Discrepancy between patient and informant**	Spending time with others		0.1 (2.6) -8, 8	-0.3 (2.3) -5, 7	0.2 (2.7) -8, 7	0.4 (2.8) -5, 8
	Communicating with others		1.1 (3.7) -8, 11	0.8 (3.5) -7, 8	0.5 (4.1) -8, 11	2.2 (3.0) -4, 9
	Sensitivity to others		1.6 (3.2) -9, 12	1.2 (2.2) -3, 7	1.8 (3.8) -8, 12	1.5 (3.1) -9, 6
	Total		2.8 (5.5) -10, 15	1.7 (4.7) -8, 11	2.6 (5.8) -10, 14	4.1 (5.7) -8, 15
			**n %**	**n %**	**n %**	**n %**
**Discrepancy between patient and informant**	Spending time with others	Underestimate	28 25.9	9 31.0	12 24.5	7 23.3
		Congruent	49 45.4	13 44.8	21 42.9	15 50.0
		Overestimate	31 28.7	7 24.1	16 32.7	8 26.7
	Communicating with others	Underestimate	23 21.3	6 20.7	14 28.6	3 10.0
		Congruent	38 35.2	13 44.8	15 30.6	10 33.3
		Overestimate	47 43.5	10 34.5	20 40.8	17 56.7
	Sensitivity to others	Underestimate	13 12.0	2 6.9	8 16.3	3 10.0
		Congruent	43 39.8	16 55.2	18 36.7	9 30.0
		Overestimate	52 48.2	11 37.9	23 46.9	18 60.0
	Total	Underestimate	30 27.8	9 31.0	16 32.7	5 16.7
		Congruent	18 16.7	6 20.7	5 10.2	7 23.3
		Overestimate	60 55.6	14 48.3	28 57.1	18 60.0

Key: max = maximum, min = minimum, SD = standard deviation

Supplementary Table 3: SF-DEM score discrepancies between patient and informant ratings, by dementia subtype

		Alzheimer's disease (n = 56)	Dementia with Lewy bodies (n = 17)	Vascular dementia (n = 5)	Unspecified dementia (n = 30)
		**mean (SD)**	**Mean (SD)**	**Mean (SD)**	**Mean (SD)**
		**min, max**	**min, max**	**min, max**	**min, max**
**Discrepancy between patient and informant**	Spending time with others	0.5 (2.9)	-0.5 (2.4)	0.4 (1.9)	-0.2 (2.3)
		-8, 8	-5, 4	-3, 2	-5, 7
	Communicating with others	1.3 (3.8)	-0.4 (3.9)	4.0 (2.3)	0.9 (3.5)
		-8, 11	-6, 7	1, 6	-7, 8
	Sensitivity to others	1.7 (3.8)	1.2 (2.9)	3.4 (3.2)	1.2 (2.3)
		-9, 12	-5, 6	-2, 6	-3, 7
	Total	3.5 (5.8)	0.4 (5.1)	7.8 (4.8)	1.9 (4.7)
		-10, 15	-7, 11	0, 13	-10, 15

Supplementary Table 4: Association of awareness into social functioning with Mini-Mental Status Examination domain performance

			**Model 1: unadjusted**			**Model 2: adjusted for age, sex, living alone, site**		
			**Coefficient**	**95% CI**	**p-value**	**Coefficient**	**95% CI**	**p-value**
**RECALL**	Number of SF-DEM points overestimation by patient per **one** point worse MMSE domain performance	**Spending time with others**	-0.2	-0.6, 0.3	0.50	-0.1	-0.6, 0.4	0.71
		**Communicating with others**	0.2	-0.5, 0.8	0.65	0.1	-0.6, 0.9	0.75
		**Sensitivity to others**	0.3	-0.3, 0.9	0.27	0.3	-0.4, 0.9	0.42
		**SF-DEM Total**	0.3	-0.7, 1.3	0.53	0.3	-0.8, 1.4	0.60
**ATTENTION / CALCULATION**	Number of SF-DEM points overestimation by patient per **one** point worse MMSE domain performance	**Spending time with others**	0.2	-0.1, 0.5	0.28	0.2	-0.1, 0.6	0.17
		**Communicating with others**	-0.1	-0.5, 0.3	0.62	-0.1	-0.6, 0.3	0.63
		**Sensitivity to others**	0.2	-0.2, 0.6	0.28	0.3	-0.1, 0.6	0.20
		**SF-DEM Total**	0.3	-0.4, 0.9	0.42	0.4	-0.3, 1.0	0.26

Key: CI = confidence interval; MMSE = mini-mental status examination; SF-DEM = Social functioning in dementia scale
